# A GSK3-SRF Axis Mediates Angiotensin II Induced Endothelin Transcription in Vascular Endothelial Cells

**DOI:** 10.3389/fcell.2021.698254

**Published:** 2021-07-26

**Authors:** Yuyu Yang, Huidi Wang, Hongwei Zhao, Xiulian Miao, Yan Guo, Lili Zhuo, Yong Xu

**Affiliations:** ^1^Jiangsu Key Laboratory for Medical Biotechnology, College of Life Sciences, Nanjing Normal University, Nanjing, China; ^2^Institute of Biomedical Research, Liaocheng University, Liaocheng, China; ^3^Key Laboratory of Targeted Intervention of Cardiovascular Disease and Collaborative Innovation Center for Cardiovascular Translational Medicine, Department of Pathophysiology, Nanjing Medical University, Nanjing, China; ^4^College of Life Sciences, Liaocheng University, Liaocheng, China; ^5^Department of Geriatrics, The Second Affiliated Hospital of Nanjing Medical University, Nanjing, China

**Keywords:** transcriptional regulation, vascular endothelial cell, post-translational modification, phosphorylation, serum response factor

## Abstract

Endothelin, encoded by *ET1*, is a vasoactive substance primarily synthesized in vascular endothelial cells (VECs). Elevation of endothelin levels, due to transcriptional hyperactivation, has been observed in a host of cardiovascular diseases. We have previously shown that serum response factor (SRF) is a regulator of *ET1* transcription in VECs. Here we report that angiotensin II (Ang II) induced *ET1* transcription paralleled activation of glycogen synthase kinase 3 (GSK3) in cultured VECs. GSK3 knockdown or pharmaceutical inhibition attenuated Ang II induced endothelin expression. Of interest, the effect of GSK3 on endothelin transcription relied on the conserved SRF motif within the *ET1* promoter. Further analysis revealed that GSK3 interacted with and phosphorylated SRF at serine 224. Phosphorylation of SRF by GSK3 did not influence its recruitment to the *ET1* promoter. Instead, GSK3-mediated SRF phosphorylation potentiated its interaction with MRTF-A, a key co-factor for SRF, which helped recruit the chromatin remodeling protein BRG1 to the *ET1* promoter resulting in augmented histone H3 acetylation/H3K4 trimethylation. Consistently, over-expression of a constitutively active GSK enhanced Ang II-induced *ET1* transcription and knockdown of either MRTF-A or BRG1 abrogated the enhancement of *ET1* transcription. In conclusion, our data highlight a previously unrecognized mechanism that contributes to the transcriptional regulation of endothelin. Targeting this GSK3-SRF axis may yield novel approaches in the intervention of cardiovascular diseases.

## Introduction

The vascular endothelium not only functions as a physical barrier separating the circulation from the basal lamina but synthesizes and secretes a host of substances contributing to the maintenance and regulation of internal microenvironment either locally (via paracrine) or systemically (via endocrine) (Rafii et al., 2016). Endothelin, encoded by *ET1*, is a polypeptide derived from endothelial cells (Yanagisawa et al., 1988). Endothelin primarily signals through one of the two cognate receptors, ETRA and ETRB. Whereas binding of endothelin to ETRA leads to contraction of vascular smooth muscle cells, the endothelin-ETRB interaction is thought to trigger endothelin clearance and vasorelaxation (Abman, 2009). Elevated plasma endothelin levels have been observed in patients with hypertension (Letizia et al., 1997), pulmonary hypertension (Rubens et al., 2001), atherosclerosis (Hasdai et al., 1997), diabetes (Cardillo et al., 2002), and heart failure (Wei et al., 1994). Concordantly, ETR antagonists have been demonstrated to be effective in the intervention of a host of cardiovascular and metabolic diseases (Enevoldsen et al., 2020).

Increased production of endothelin is primarily achieved through transcriptional activation of the *ET1* gene, which is localized on chromosome 6 in the human genome (Stow et al., 2011). The proximal *ET1* promoter is responsive to a number of stimuli including angiotensin II (Ang II), hypoxia, transforming growth factor (TGF), thrombin, and tumor necrosis factor (TNF) (Imai et al., 1992; Delerive et al., 1999; Yamashita et al., 2001; Castanares et al., 2007; Wort et al., 2009). The stimulatory effects of these humoral factors on *ET1* transcription are mediated by different sequence-specific transcription factors. For instance, we and others have shown that activator protein 1 (AP-1) is recruited to the -115/-108 region of the *ET1* promoter in response to Ang II and mediates *ET1* trans-activation in endothelial cells (Lee et al., 1991; Imai et al., 1992; Weng et al., 2015a). Hypoxia-induced ET1 trans-activation can be mediated by, according to Yamashita et al. (2001), a complex that contains HIF-1α, AP-1, and GATA-2. Alternatively, we have previously shown that serum response factor (SRF) can be detected on the proximal *ET1* promoter and mediates hypoxia induced *ET1* trans-activation (Yang et al., 2013).

In the vasculature, SRF is known to regulate the transcription of contractile genes in vascular smooth muscle cells by recognizing the so-called CArG box [CC(A/T)_6_GG] located on its target promoters (Miano, 2010). The role of SRF in the regulation of endothelial function is relatively less well defined. It has been demonstrated independently by Franco et al. (2008) and by Holtz et al. (Holtz and Misra, 2008) that endothelium-specific deletion of SRF in mice results in embryonic lethality owing to defective angiogenesis/vasculogenesis and hemorrhaging. Transcrptionally, SRF is essential for the activation of E-Cadherin (*CDH5*) and β-actin (*ACTAB*) to maintain endothelial homeostasis. The transcriptional activity of SRF is subject to multiple layers of modulation including post-translational modifications (PTMs). Matsuzaki et al. (2003), for instance, have discovered that lysine residue 147 of SRF can be SUMOylated, which suppresses its transcriptional activity likely by interfering with its DNA binding potential. More recently, Kwon et al. (2021) have reported that lysine residue 165 of SRF can be methylated by SET7 and de-methylated by KDM2B; dynamic SRF methylation is proposed to regulate its affinity for target promotes and contribute to muscle differentiation. By far, phosphorylation represents the most extensively characterized PTM for SRF. There is evidence to support a host of kinases, including MK2 (Heidenreich et al., 1999), PKA (Blaker et al., 2009), CaMKII (Ely et al., 2011), GSK3β (Li et al., 2014), and PKC (Iyer et al., 2006), using SRF as a substrate. Whether or not SRF phosphorylation can contribute to *ET1* transcription is yet to be examined. Here we report that GSK3β mediated SRF phosphorylation facilitates its interaction with key co-factors to mediate Ang II induced endothelin expression in endothelial cells.

## Materials and Methods

### Cell Culture

Immortalized human endothelial cells (EAhy926) were maintained in DMEM supplemented with 10% FBS as previously described (Chen et al., 2020c). ET1 promoter-luciferase constructs (Yang et al., 2013), SRF expression constructs (Li et al., 2014; Kong et al.,2019a,b) have been previously described. Constitutively active GSK3β, in which serine 9 was substituted by alanine (S9A), dominant negative GSK3β, in which arginine 96 was substituted by alanine (R96A), phosphorylation-defective SRF, in which serine 224 was substituted by alanine (S224A), and phosphorylation mimetic SRF in which serine 224 was substituted by aspartic acid (S224D), were generated by a QuikChange kit (Thermo Fisher Scientific, Waltham, MA, United States) and verified by direct sequencing as previously described (Bechard and Dalton, 2009). Angiotensin II was purchased from Sigma (St. Louis, MO, United States). LY2090314 were purchased from Selleck (Houston, TX, United States). Small interfering RNAs were purchased from Dharmacon (Lafayette, CO, United States): siGSK3B#1, AAGTAATCCACCTCTGGCTAC; siGSK3B#2, GUAAUCCACCUCUGGCUAC; siMRTFA#1, GUG UCUUGGUGUAGUGU; siMRTFA#2, CUGCGUGCAU AUCAAGAAC; siBRG1#1, AACATGCACCAGATGCAC AAG; siBRG1#2, GCCCATGGAGTCCATGCAT. Transient transfections were performed with Lipofectamine 2000 (for DNA plasmids, Thermo Fisher Scientific, Waltham, MA, United States) or Lipofectamine RNAiMax (for siRNAs, Thermo Fisher Scientific, Waltham, MA, United States). Cells were harvested 48 h after transfection and reporter activity was measured using a luciferase reporter assay system (Promega, Madison, WI, United States) as previously described (Wu X. et al., 2020; Hong et al., 2021).

### Protein Extraction, Immunoprecipitation, and Western Blotting

Whole cell lysates were obtained by re-suspending cell pellets in RIPA buffer (50 mM Tris pH7.4, 150 mM NaCl, 1% Triton X-100) with freshly added protease inhibitor (Roche, Basel, Switzerland) as previously described (Chen et al.,2020a,b, c, 2021; Dong et al., 2020, 2021; Fan et al., 2020; Li N. et al., 2020; Li et al.,2020a,b; Lv et al., 2020; Mao et al., 2020; Sun et al., 2020; Wu T. et al., 2020; Wu X. et al., 2020; Yang et al., 2020; Hong et al., 2021; Kong et al., 2021; Zhang et al., 2021). Specific antibodies were added to and incubated with cell lysates overnight before being absorbed by Protein A/G-plus Agarose beads (Santa Cruz, Santa Cruz, CA, United States). Precipitated immune complex was released by boiling with 1X SDS electrophoresis sample buffer. Proteins were separated by 8% polyacrylamide gel electrophoresis with pre-stained markers (Bio-Rad, Hercules, CA, United States) for estimating molecular weight and efficiency of transfer to blots. Proteins were transferred to nitrocellulose membranes (Bio-Rad, Hercules, CA, United States) in a Mini-Trans-Blot Cell (Bio-Rad, Hercules, CA, United States). The membranes were blocked with 5% milk powder in Tris-buffered saline buffer (0.05% Tween 20, 150 mM NaCl, 100 mM Tris-HCl pH7.4) at 4°C overnight. Western blot analyses were performed with anti-GFP (Proteintech, 50430-2, Wuhan, China), anti-FLAG (Sigma, F3165, St. Louis, MO, United States), anti-GSK3β (Cell Signaling Tech, 12456, Danvers, MA, United States), anti-phospho-Ser9-GSK3β (Cell Signaling Tech, 5558), anti-GSK3α (Proteintech, 13419-1, Wuhan, China), anti-phospho-Ser21-GSK3α (Cell Signaling Tech, 9316, Danvers, MA, United States), anti-phospho-(S/T)F (Cell Signaling Tech, 9631, Danvers, MA, United States), anti-SRF (Cell Signaling Tech, 5147, Danvers, MA, United States), and anti-β-actin (Sigma, A2228, St. Louis, MO, United States) antibodies. For densitometrical quantification, densities of target proteins were normalized to those of β-actin as previously described (Sun et al., 2020; Wu T. et al., 2020). Data are expressed as relative protein levels compared to the control group which is arbitrarily set as 1.

### RNA Isolation and Real-Time PCR

RNA was extracted with the RNeasy RNA isolation kit (Qiagen, Hilden, Germany). Reverse transcriptase reactions were performed using a SuperScript First-strand Synthesis System (Invitrogen, Waltham, MA, United States) as previously described (Zhang et al., 2020; Liu et al., 2021). Real-time PCR reactions were performed on an ABI Prism 7500 system with the following primers: *ET1*, 5′-AGAGTGTGTCTACTTCTGCCA-3′ and 5′-CTTCCAAGTCCATACGGAACAA-3′; GSK3B, 5′-GG CAGCATGAAAGTTAGCAGA-3′ and 5′-GGCGACCAGT TCTCCTGAATC-3′. Ct values of target genes were normalized to the Ct values of housekeeping control gene (18s, 5′-CGCGGTTCTATTTTGTTGGT-3′ and 5′-TCGTCTTCGAAACTCCGACT-3′ for both human and mouse genes) using the ΔΔCt method and expressed as relative mRNA expression levels compared to the control group which is arbitrarily set as 1.

### Chromatin Immunoprecipitation (ChIP)

Chromatin immunoprecipitation (ChIP) assays were performed essentially as described before. In brief, chromatin in control and treated cells were cross-linked with 1% formaldehyde. Cells were incubated in lysis buffer (150 mM NaCl, 25 mM Tris pH 7.5, 1% Triton X-100, 0.1% SDS, 0.5% deoxycholate) supplemented with protease inhibitor tablet and PMSF. DNA was fragmented into ∼200 bp pieces using a Branson 250 sonicator (Brookfield, CT, United States). Aliquots of lysates containing 200 μg of protein were used for each immunoprecipitation reaction with anti-MRTF-A (Santa Cruz, sc-10768), anti-SRF (Cell Signaling Tech, 5147, Danvers, MA, United States), anti-BRG1 (Abcam, ab110641, Cambridge, United Kingdom), anti-acetyl histone H3 (Millipore, 06-599, Burlington, MA, United States), and anti-trimethyl H3K4 (Millipore, 07-442, Burlington, MA, United States). Precipitated genomic DNA was amplified by real-time PCR with the following primers: *ET1* proximal promoter, 5′-GGCGTCTGCCTCTGAAGT-3′ and 5′-GGGTAAACAGCTCCGACTT-3′. A total of 10% of the starting material is also included as the input. Data are then normalized to the input and expressed as% recovery relative the input as previously described (Chen et al.,2020b,c). All experiments were performed in triplicate wells and repeated three times.

### Enzyme-Linked Immunosorbent Assay (ELISA)

Secreted endothelin in the culture media was measured by ELISA using a commercially available kit (R&D, DET100, Minneapolis, MN, United States) per vendor recommendations.

### Statistical Analysis

Sample sizes reflected the minimal number needed for statistical significance based on power analysis and prior experience. Two-tailed Student’s *t*-test was performed using an SPSS package. Unless otherwise specified, *p*-values smaller than 0.05 were considered statistically significant.

## Results

### Ang II Induced Endothelin Expression Parallels GSK3 Activation

**FIGURE 1:**
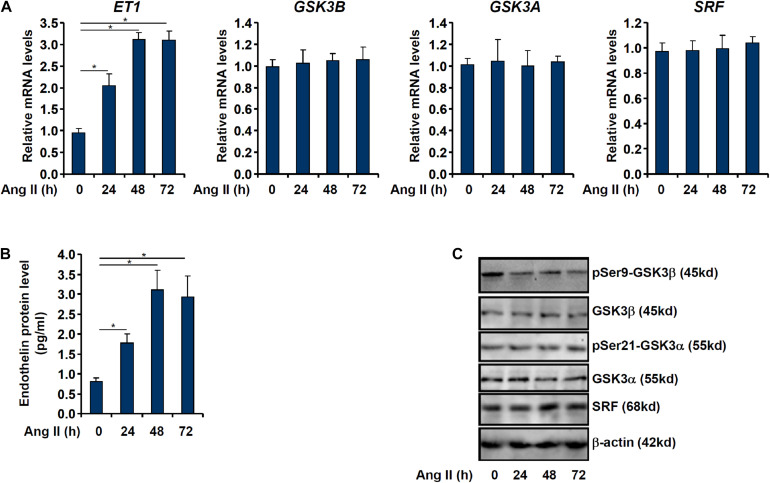
Ang II induced endothelin expression parallels GSK3 activation. EAhy926 cells were treated with Ang II (0.1 μM) and harvested at indicated time points. Endothelin expression levels were examined by qPCR **(A)** and ELISA **(B)**. GSK3 phosphorylation was examined by Western **(C)**. Error bars represent SD (*p < 0.05, two-way Student’s *t*-test). All experiments were repeated three times and one representative experiment is shown.

When cultured endothelial cells were exposed to Ang II treatment, ET1 expression levels were significantly up-regulated as measured by qPCR (Figure 1A) and ELISA (Figure 1B) in keeping with previous findings (Weng et al.,2015a,b; Yu et al., 2015). Of note, although GSK3β expression levels were not altered by Ang II treatment, GSK3β activity, as measured by loss of serine 9 phosphorylation, was up-regulated by Ang II treatment (Figures 1A,C). GSK3α is the other GSK3 isoform that shares similar structure with GSK3β but possesses distinct functions (Liang and Chuang, 2006). By comparison, neither expression nor activity of GSK3α was altered by Ang II treatment (Figures 1A,C).

### GSK3 Is Essential for Ang II Induced Endothelin Expression

**FIGURE 2:**
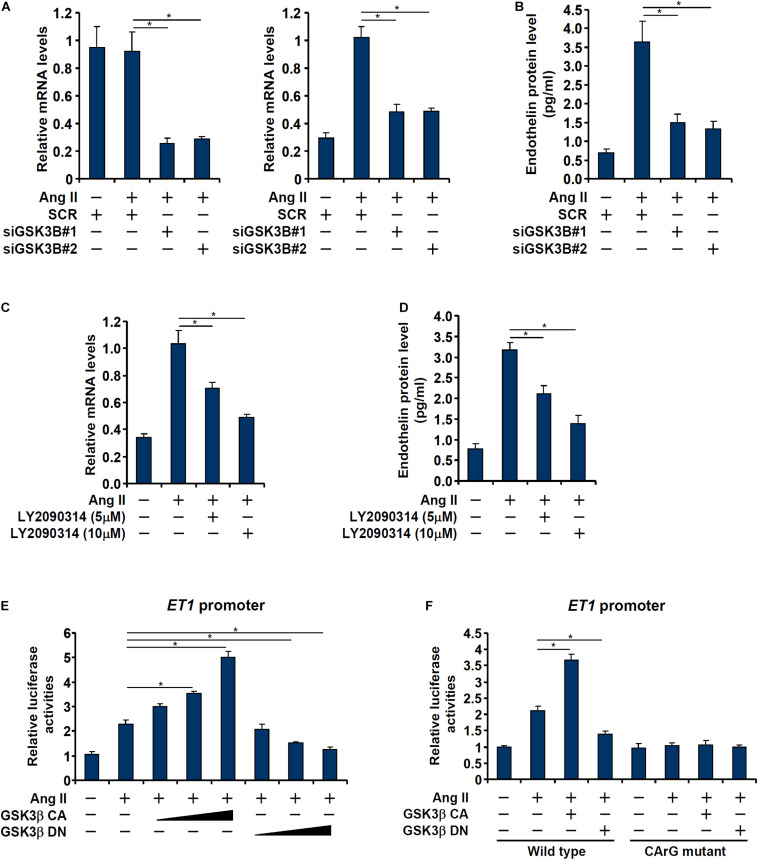
GSK3 is essential for Ang II induced endothelin expression. **(A,B)** EAhy926 cells were transfected with siRNAs targeting GSK3β or scrambled siRNA (SCR) followed by treatment with Ang II (0.1 μM) for 48 h. Endothelin expression was examined by qPCR and ELISA. **(C,D)** EAhy926 cells were treated with Ang II (0.1 μM) with or without LY2090314 for 48 h. Endothelin expression was examined by qPCR and ELISA. **(E)** An *ET1* promoter-luciferase construct was transfected into EAhy926 cells with indicated GSK3 expression constructs followed by treatment with Ang II (0.1 μM) for 48 h. Luciferase activities were normalized by protein concentration and GFP fluorescence. **(F)** Wild type or mutant *ET1* promoter-luciferase construct was transfected into EAhy926 cells with indicated GSK3 expression constructs followed by treatment with Ang II (0.1 μM) for 48 h. Luciferase activities were normalized by protein concentration and GFP fluorescence. Error bars represent SD (*p < 0.05, two-way Student’s *t*-test). All experiments were repeated three times and one representative experiment is shown.

We asked whether GSK3 activation and endothelin expression was correlative or causative. In the first set of experiments, endogenous GSK3 was depleted with two separate pairs of siRNAs; GSK3 knockdown markedly attenuated Ang II induced endothelin expression (Figures 2A,B). In the second set of experiments, a specific GSK3 inhibitor (LY2090314) was added to the cells in the presence of Ang II; GSK3 inhibition by LY2090314 ameliorated endothelin induction in a dose-dependently manner (Figures 2C,D).

Next, we asked whether regulation of endothelin expression by GSK3 occurred at the transcriptional level. To this end, an *ET1* promoter-luciferase construct was transfected into EAhy926 cells with or without a constitutively active (CA) GSK3β (S9A) or a dominant negative (DN) GSK3β (S9D). As shown in Figure 2E, over-expression of GSK3β CA enhanced the activation of the *ET1* promoter whereas over-expression of GSK3β DN dampened the activation of the *ET1* promoter. Interestingly, the effect of GSK3β on the *ET1* promoter seemed to rely on the SRF site located between −48 and −41 relative to the transcription start site (Yang et al., 2013) as mutagenesis of this site completely abrogated the response of the *ET1* promoter to GSK3β over-expression (Figure 2F).

### GSK3 Interacts With and Phosphorylates SRF in Endothelial Cells

**FIGURE 3:**
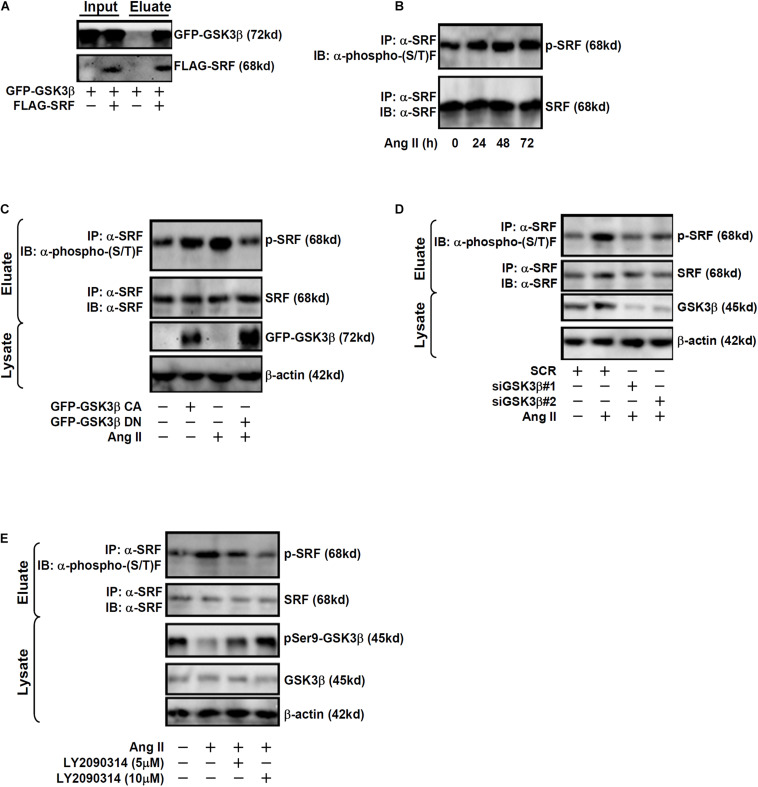
GSK3 interacts with and phosphorylates SRF in endothelial cells. **(A)** EAhy926 cells were transduced with FLAG-tagged SRF and GFP-tagged GSK3β followed by treatment with Ang II (0.1 μM). Immunoprecipitation was performed with anti-FLAG. **(B)** EAhy926 cells were treated with Ang II (0.1 μM) and harvested at indicated time points. Immunoprecipitation was performed with anti-SRF. **(C)** EAhy926 cells were transduced with adenovirus carrying GFP-tagged GSK3β CA or GSK3β DN followed by treatment with Ang II (0.1 μM). Immunoprecipitation was performed with anti-SRF. **(D)** EAhy926 cells were transfected with siRNAs targeting GSK3β or scrambled siRNA (SCR) followed by treatment with Ang II (0.1 μM) for 48 h. Immunoprecipitation was performed with anti-SRF. **(E)** EAhy926 cells were treated with Ang II (0.1 μM) with or without LY2090314 for 48 h. Immunoprecipitation was performed with anti-SRF.

Previously, Li et al. (2014) have reported that SRF can be phosphorylated by GSK3β in neurons to promote axonal growth. Immunoprecipitation assay was performed to verify GSK3β could interact with SRF in endothelial cells. EAhy926 cells were transduced with adenovirus carrying FLAG-tagged SRF and/or GFP-tagged GSK3β. An anti-FLAG could precipitate both SRF and GSK3β only when both were over-expressed thus confirming that SRF and GSK3β could interact with each other in endothelial cells (Figure 3A). Although Ang II treatment did not affect SRF expression levels in endothelial cells (Figures 1A,C), it significantly up-regulated SRF phosphorylation levels (Figure 3B). Based on these observations, we hypothesized that GSK3β might phosphorylate SRF to participate in ET1 transcription. As shown in Figure 3C, over-expression of GFP-tagged GSK3β CA alone was sufficient to augment SRF phosphorylation in the absence of Ang II whereas over-expression of GFP-tagged GSK3β DN suppressed Ang II-induced SRF phosphorylation. In addition, knockdown of endogenous GSK3β by siRNAs or GSK3β inhibition by a small-molecule compound (LY2090314) comparably attenuated SRF phosphorylation by Ang II treatment (Figures 3D,E).

### Phosphorylation of SRF by GSK3 Is Essential for Ang II Induced Endothelin Expression

**FIGURE 4:**
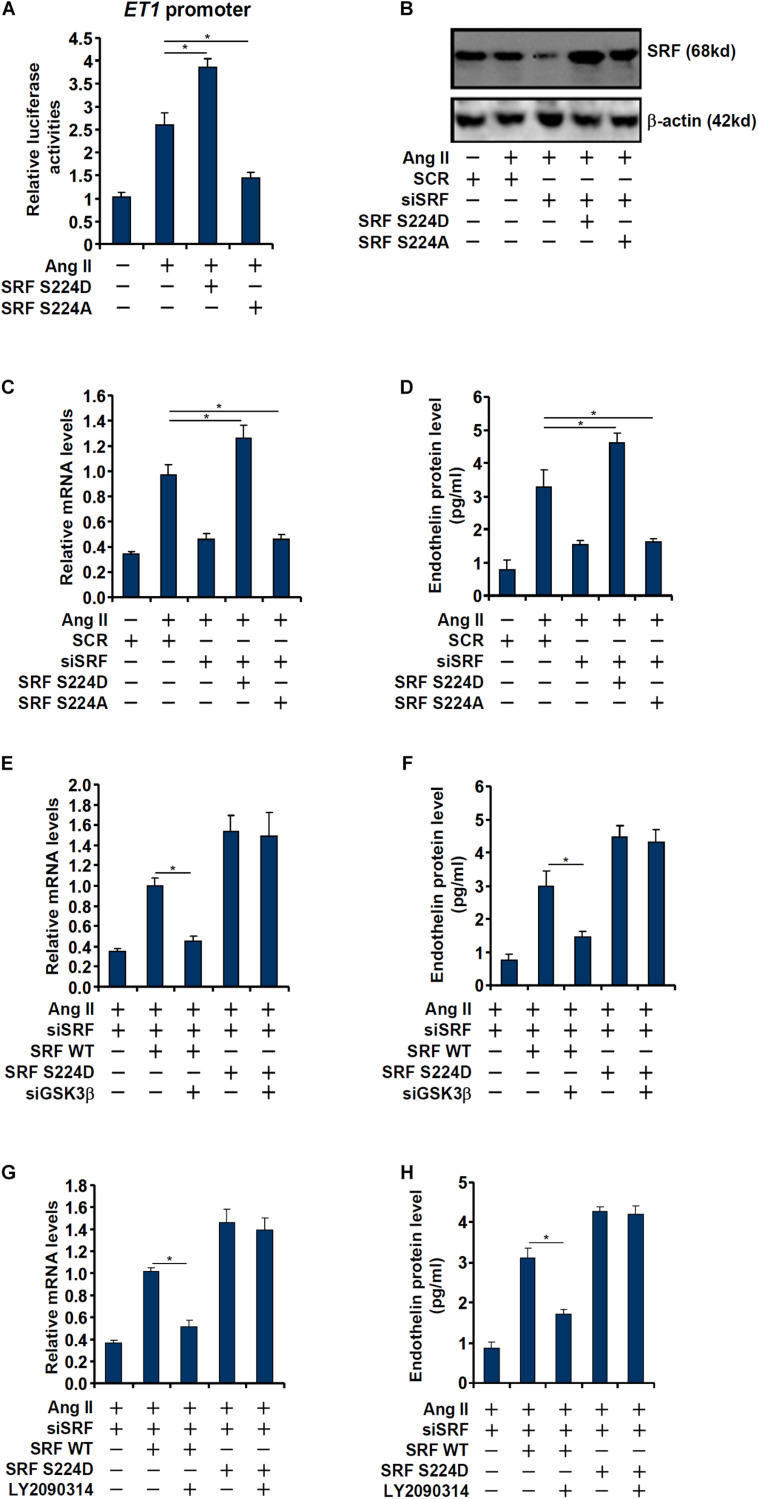
Phosphorylation of SRF by GSK3 is essential for Ang II induced endothelin expression. **(A)** An *ET1* promoter-luciferase construct was transfected into EAhy926 cells with indicated SRF expression constructs (S224A or S224D) followed by treatment with Ang II (0.1 μM) for 48 h. Luciferase activities were normalized by protein concentration and GFP fluorescence. **(B–D)** EAhy926 cells were transfected with siRNA targeting SRF followed by transduction with adenoviral SRF expression constructs (S224A or S224D) and treatment with Ang II (0.1 μM) for 48 h. SRF expression was examined by Western. Endothelin expression was examined by qPCR and ELISA. **(E,F)** EAhy926 cells were transfected with siRNA targeting SRF/GSK3β followed by transduction with adenoviral SRF expression constructs (WT or S224D) and treatment with Ang II (0.1 μM) for 48 h. Endothelin expression was examined by qPCR and ELISA. **(G,H)** EAhy926 cells were transfected with siRNA targeting SRF followed by transduction with adenoviral SRF expression constructs (WT or S224D) and treatment with Ang II (0.1 μM) and LY2090314 for 48 h. Endothelin expression was examined by qPCR and ELISA. Error bars represent SD (*p < 0.05, two-way Student’s *t*-test). All experiments were repeated three times and one representative experiment is shown.

Next, we evaluated the relevance of GSK3β-mediated SRF phosphorylation in ET1 transcription. In the reporter assay, it was observed that over-expression of a phosphomimetic SRF (S234D) enhanced trans-activation of the ET1 promoter by Ang II whereas over-expression of a phosphor-defective SRF (S234A) weakened ET1 trans-activation (Figure 4A). Next, endogenous SRF in endothelial cells was depleted by siRNAs followed by re-introduction of ectopic SRF via adenviral transduction (Figure 4B). As shown in Figures 4C,D, whereas SRF depletion attenuated Ang II induced ET1 expression, introduction of SRF S234D more than compensated for the loss of endogenous SRF by restoring ET1 induction; SRF S234A, however, failed to recover the reduction of ET1 expression. In the next set of experiments, wild type (WT) or the phosphomimetic (S234D) SRF was over-expressed in SRF-depleted endothelial cells followed by GSK3β knockdown or inhibition. Whereas GSK3β knockdown or inhibition completely deprived the ability of WT SRF to rescue ET1 expression in SRF-depleted endothelial cells, the phosphomimetic SRF was refractory to the manipulation of GSK3β expression/activity (Figures 4E–H). Taken together, these data point to an interplay between SRF and GSK3β that contributes to Ang II induced ET1 trans-activation.

### Phosphorylation of SRF by GSK3 Is Essential for Co-factor Recruitment

**FIGURE 5:**
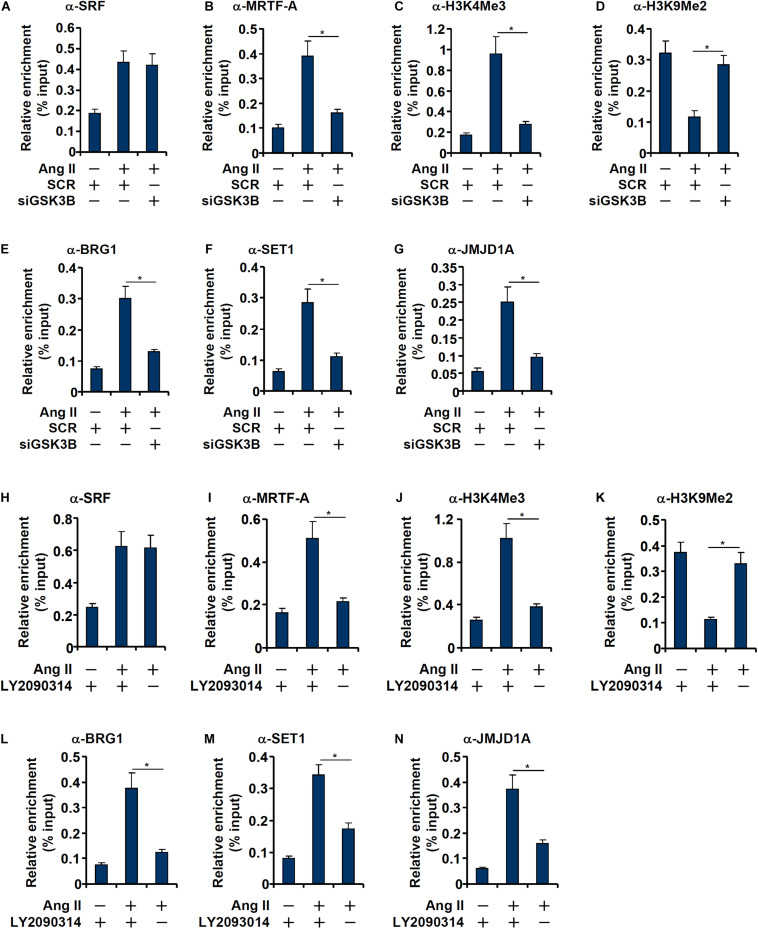
GSK3 knockdown or inhibition attenuates recruitment of co-factors to the ET1 promoter. **(A–G)** EAhy926 cells were transfected with siRNAs targeting GSK3β or scrambled siRNA (SCR) followed by treatment with Ang II (0.1 μM) for 48 h. ChIP assays were performed with anti-SRF, anti-MRTF-A, anti-H3K4Me3, anti-H3K9Me2, anti-BRG1, anti-SET1, and anti-JMJD1A. **(H–N)** EAhy926 cells were treated with Ang II (0.1 μM) with or without LY2090314. ChIP assays were performed with anti-SRF, anti-MRTF-A, anti-H3K4Me3, anti-H3K9Me2, anti-BRG1, anti-SET1, and anti-JMJD1A. Error bars represent SD (*p < 0.05, two-way Student’s *t*-test). All experiments were repeated three times and one representative experiment is shown.

Consistent with our prior observation (Yang et al., 2013), chromatin immunoprecipitation (ChIP) assay showed that Ang II treatment significantly enhanced the occupancies of SRF (Figure 5A) and MRTF-A (Figure 5B), a key co-activator of SRF, on the ET1 promoter. GSK3β depletion did not affect SRF binding (Figure 5A) but markedly dampened the binding of MRTF-A (Figure 5B). Typically, active transcription is associated with enrichment of trimethylated H3K4 (H3K4Me3) on the gene promoters (Shilatifard, 2012). On the contrary, transcriptional repression can be measured by enrichment of dimethylated H3K9 (H3K9Me2) on the gene promoters (Piunti and Shilatifard, 2021). Previous studies have shown that SRF interacts with MRTF-A to regulate transcription by differentially modulating histone methylation levels (Kong et al.,2019a,b). ChIP assays showed that Ang II treatment triggered an increase in H3K4Me3 (Figure 5C) and a simultaneous decrease in H3K9Me2 (Figure 5D) on the ET1 promoter, both of which were reversed by GSK3β depletion. Consistently, the epigenetic factors involved in catalyzing these characteristic histone modifications, namely BRG1 (Figure 5E), SET1 (Figure 5F), and JMJD1A (Figure 5G), were similarly recruited to the ET1 promoter upon Ang II treatment but disrupted by GSK3β knockdown. In the second set of experiments, LY2090314 was added to the endothelial cells to inhibit GSK3β activity: GSK3b inhibition similarly led to dampened recruitment of MRTF-A (Figure 5I), BRG1 (Figure 5L), SET1 (Figure 5M), and JMJD1A (Figure 5N), erasure of trimethyl H3K4 (Figure 5J), and accumulation of dimethyl H3K9 (Figure 5K) without influencing SRF binding (Figure 5H) to the ET1 promoter.

**FIGURE 6:**
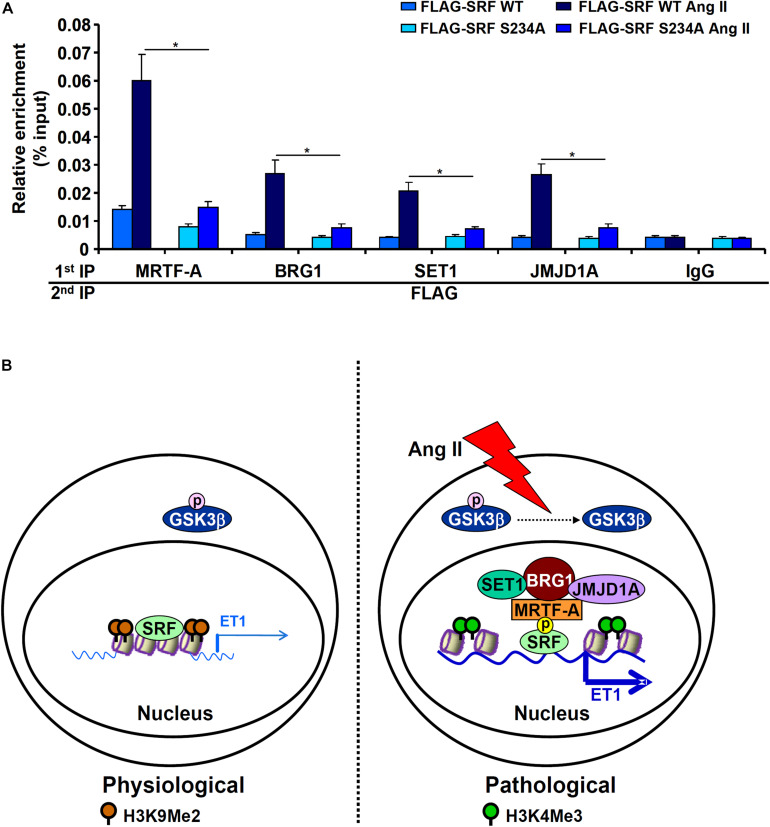
GSK3-mediated SRF phosphorylation licenses the assembly a transcriptional complex to activate endothelin expression. **(A)** EAhy926 cells were transfected with siRNA targeting SRF followed by transduction with adenoviral SRF expression constructs and treatment with Ang II (0.1 μM) for 48 h. Re-ChIP assays were performed with indicated antibodies. Error bars represent SD (*p < 0.05, two-way Student’s *t*-test). All experiments were repeated three times and one representative experiment is shown. **(B)** A schematic model.

Next, we performed Re-ChIP assay to evaluate whether GSK3-mediated SRF phosphorylation might be necessary for the assembly of a transcriptional complex on the ET1 promoter. As shown in Figure 6A, in the presence of WT SRF, Ang II treatment stimulated the assembly of an SRF-centered complex that contained MRTF-A, BRG1, SET1, and JMJD1A. When replaced by a phosphorylation-defective SRF (S234A), incorporation of co-factors into this transcriptional complex was severely compromised. Taken together, these data support a model wherein GSK3-mediated SRF phosphorylation licenses the assembly of a transcriptional complex to activate endothelin expression (Figure 6B).

## Discussion

Post-translational modifications play a key role in the regulation of cardiovascular diseases (Smith and White, 2014; Gajjala et al., 2015; Fert-Bober et al., 2018). Alteration of SRF activity contributes to the disruption of cardiovascular homeostasis and is associated with a wide range of cardiovascular diseases (Miano, 2010). SRF relies on its interaction with co-factors to orchestrate specific transcriptional events and participate in disease pathogenesis (Onuh and Qiu, 2020). Based on our findings here, we propose a model in which phosphorylation of SRF by GSK3β mediates Ang II induced *ET1* transcription (Figure 6B).

We show here that GSK3β is activated by Ang II treatment in endothelial cells. This observation is consistent with a previous report by Zhang et al. (2017) showing that Ang II treatment activates GSK3β in human primary umbilical endothelial cells. Similarly, blockade of Ang II signaling by a specific angiotensin II receptor antagonist Telmisartan inhibits GSK3β activity in endothelial cells (Song et al., 2017). On the contrary, Ang II infusion seems to have an inhibitory effect on GSK3β in cardiomyocytes (Li et al., 2005) and smooth muscle cells (Cuevas et al., 2015). One possible explanation could be that differential expression levels of angiotensin receptor 1 (AT1) and 2 (AT2) in these cells because AT1 and AT2 often exert opposing roles once engaged by angiotensin II (Kaschina et al., 2017).

Our data suggest that GSK3β may contribute to ET1 transcription via SRF phosphorylation. These data, however, leave the door open to alternative interpretations. First, SRF phosphorylation status is influenced by multiple kinases other than GSK3β. For instance, SRF can be phosphorylated by MK2 at serine residue 103, which enhances its DNA binding activity (Heidenreich et al., 1999). Because MK2 can be placed downstream of Ang II and MK2 completely abrogates Ang II induced organ damage *in vivo* (Bao et al., 2007), it is plausible that Ang II may induce ET1 transcription through MK2-mediated SRF phosphorylation. Alternatively, phosphorylation of SRF at threonine residue 160 by CaMKII potentiates its DNA binding (Fluck et al., 2000). Mounting evidences suggests that CaMKII signaling plays an essential role mediating the pathophysiological effects of Ang II (Li et al., 2010; Prasad et al., 2015; Basu et al., 2019). It is reasonable to speculate that the ability of SRF to trans-activate ET1 expression may be attributable, at least in part, to CaMKII-catalyzed T160 phosphorylation. Second, other components of the SRF-centered transcriptional complex may be subject to GSK3β mediated phosphorylation. The Treisman laboratory has previously systemically profiled the phosphorylation status of MRTF-A in MEF cells with the finding that several serine/threonine residues may be targeted by GSK3β; collective mutation of these sites blocks nuclear trans-location of MRTF-A (Panayiotou et al., 2016). BRG1, SET1, and JMJD1A all have been demonstrated to be phosphorylated under specific circumstances (Cheng et al., 2014; Padilla-Benavides et al., 2017) although it remains to be determined whether GSK3β makes a contribution.

There are a few unsolved issues that warrant further investigation. First, we focused in the present study on the effect of SRF S234 phosphorylation on ET1 transcription. It is not clear how genomewide gene expression levels would be affected by this specific modification. A recent study by Li et al. have shown that dynamic regulation of SRF serine 103 phosphorylation by RSK3 and PP2A significantly impacts phenylephrine-induced gene transcription in cardiomyocytes. Specifically, the authors demonstrate, using ChIP-seq and PRO-seq, that the phosphomimetic SRF S103D appears to promote the association of SRF with enhancers, facilitate the recruitment of the basal transcription machinery, and preferentially augment the expression of early response and hypertrophic genes (Li J. et al., 2020). Similar strategies could exploited to determine how the SRF S234D mutant influence the endothelial transcriptome. Second, the functional relevance of the present study is not entirely clear. Global deletion of GSK3β exacerbates myocardial hypertrophy possibly due to hyperproliferation of cardiomyoblast (Kerkela et al., 2008). On the other hand, GSK3β hypomorphic mice are protected from dilated cardiomyopathy and heart failure (Mohamed et al., 2016). Endothelial-specific role for GSK3β is yet to be determined. Third, although our model suggests that SRF is a primary substrate for GSK3β, it does not rule out the possibility that GSK3β may indirectly regulate SRF activity. Both SRF and its most importantly cofactor MRTF-A are modulated by cytoskeletal remodeling (Olson and Nordheim, 2010). GSK3β, by targeting multiple components of cytoskeleton, is considered a key mechanosensor in endothelial cells (Gaetani et al., 2020). Therefore, it is conceivable that GSK3β may contribute to ET1 transcription by relaying the mechanic signal to the SRF complex. Finally, although we focused the effect of SRF phosphorylation on its interaction with histone methyltransferase/demethylase, the potential involvement of histone acetyltransferases/deacetylase cannot be excluded. Several histone acetyltransferases with distinct substrate specificities including PCAF (Puttagunta et al., 2014), p300 (Kong et al., 2019b), CBP (Qiu and Li, 2002), and KAT8 (Kong et al., 2019a), have been shown to interact with SRF and participate in SRF-mediated transcriptional events. Previous studies have implicated p300 and CBP in the regulation of ET1 transcription in endothelial cells (Yamashita et al., 2001; Cianfrocca et al., 2016). It would be of interest to determine whether recruitment of CBP and/or p300 might be affected by SRF phosphorylation.

## Conclusion

In conclusion, our data portray GSK3β as a regulator of Ang II-induced ET1 transcription in endothelial cells by licensing the assembly of an phospho-SRF-MRTF-A-BRG1-SET1-JMJD1A complex. Further investigations are needed to solidify the role of this complex in regulating endothelial function and eventually devise novel therapeutic strategies by targeting this complex.

## Data Availability Statement

The original contributions presented in the study are included in the article/Supplementary Material, further inquiries can be directed to the corresponding author/s.

## Author Contributions

LZ conceived the project. YY, HW, HZ, XM, and YG designed and performed the experiments, and collected and analyzed the data. YX wrote the manuscript. YY and LZ secured funding and provided supervision. All authors contributed to the article and approved the submitted version.

## Conflict of Interest

The authors declare that the research was conducted in the absence of any commercial or financial relationships that could be construed as a potential conflict of interest.

## Publisher’s Note

All claims expressed in this article are solely those of the authors and do not necessarily represent those of their affiliated organizations, or those of the publisher, the editors and the reviewers. Any product that may be evaluated in this article, or claim that may be made by its manufacturer, is not guaranteed or endorsed by the publisher.
