# Supplementary material for: A GSK3-SRF Axis Mediates Angiotensin II Induced Endothelin Transcription in Vascular Endothelial Cells
Source: Front Cell Dev Biol. 2021 Jul 26;9:698254. doi: 10.3389/fcell.2021.698254 (PMC8350349; doi:10.3389/fcell.2021.698254)

Real-time PCR performed July 30 2020
Samples prepared July 30 2020
EAhy926

1. GSK3B

sample ID	ct target	ct 18s	dct	ddct	ratio	average	SD
0h	27.45	8.19	19.26	-0.07	1.049717	0.989968	0.065345
	27.88	8.43	19.45	0.12	0.920188		
	27.64	8.31	19.33	0	1		
24h	27.19	7.95	19.24	-0.09	1.06437	1.021453	0.12304
	27.36	7.85	19.51	0.18	0.882703		
	27.35	8.18	19.17	-0.16	1.117287		
48h	27.05	7.83	19.22	-0.11	1.079228	1.048726	0.066308
	27.06	7.69	19.37	0.04	0.972655		
	27.12	7.92	19.2	-0.13	1.094294		
72h	27.74	8.66	19.08	-0.25	1.189207	1.056368	0.117521
	27.95	8.57	19.38	0.05	0.965936		
	27.99	8.68	19.31	-0.02	1.013959		

2. ET1

sample ID	ct target	ct 18s	dct	ddct	ratio	average	SD
0h	25.74	8.19	17.55	0.23	0.852635	0.962633	0.096879
	25.75	8.43	17.32	0	1		
	25.58	8.31	17.27	-0.05	1.035265		
24h	24.11	7.95	16.16	-1.16	2.234574	2.049715	0.269008
	24.37	7.85	16.52	-0.8	1.741101		
	24.38	8.18	16.2	-1.12	2.17347		
48h	23.55	7.83	15.72	-1.6	3.031433	3.105165	0.165174
	23.43	7.69	15.74	-1.58	2.989698		
	23.52	7.92	15.6	-1.72	3.294364		

72h	24.31	8.66	15.65	-1.67	3.182146	3.093085	0.214781
	24.19	8.57	15.62	-1.7	3.24901		
	24.49	8.68	15.81	-1.51	2.8481		

3. SRF

sample ID	ct target	ct 18s	dct	ddct	ratio	average	SD
0h	24.96	8.19	16.77	-0.03	1.021012	0.969952	0.071024
	25.23	8.43	16.8	0	1		
	25.28	8.31	16.97	0.17	0.888843		
24h	24.84	7.95	16.89	0.09	0.939523	0.978975	0.074025
	24.75	7.85	16.9	0.1	0.933033		
	24.89	8.18	16.71	-0.09	1.06437		
48h	24.68	7.83	16.85	0.05	0.965936	0.996446	0.101373
	24.62	7.69	16.93	0.13	0.913831		
	24.57	7.92	16.65	-0.15	1.109569		
72h	25.33	8.66	16.67	-0.13	1.094294	1.038403	0.048529
	25.36	8.57	16.79	-0.01	1.006956		
	25.46	8.68	16.78	-0.02	1.013959		

4. GSK3A

sample ID	ct target	ct 18s	dct	ddct	ratio	average	SD
0h	27.97	8.19	19.78	0.07	0.952638	1.008137	0.059983
	28.04	8.43	19.61	-0.1	1.071773		
	28.02	8.31	19.71	0	1		
24h	27.72	7.95	19.77	0.06	0.959264	1.04295	0.203136
	27.72	7.85	19.87	0.16	0.895025		
	27.54	8.18	19.36	-0.35	1.274561		
48h	27.35	7.83	19.52	-0.19	1.140764	0.997474	0.14115
	27.62	7.69	19.93	0.22	0.858565		
	27.64	7.92	19.72	0.01	0.993092		

72h	28.39	8.66	19.73	0.02	0.986233	1.038393	0.047521
	28.21	8.57	19.64	-0.07	1.049717		
	28.28	8.68	19.6	-0.11	1.079228		

July 31 2015

Sample ID	Absorbance	concentration (pg/ml)	average	SD
0h	0.672	0.91	0.81	0.08695
	0.633	0.75		
	0.634	0.76		
24h	0.825	1.50	1.76	0.24128
	0.947	1.98		
	0.902	1.81		
48h	1.332	3.49	3.11	0.49077
	1.282	3.29		
	1.094	2.56		
72h	1.135	2.72	2.93	0.52483
	1.341	3.52		
	1.089	2.54		

Real-time PCR performed Aug 22 2015
Samples prepared Aug 22 2015
EAhy926

1. GSK3B

sample ID	ct target	ct 18s	dct	ddct	ratio	average	SD
SCR	26.45	7.38	19.07	-0.13	1.094294	0.951543	0.146764
	26.61	7.09	19.52	0.32	0.80107		
	26.47	7.21	19.26	0.06	0.959264		
Ang II SCR	26.73	7.14	19.59	0.39	0.76313	0.923362	0.138809
	26.55	7.35	19.2	0	1		
	26.56	7.37	19.19	-0.01	1.006956		
Ang II siGSK3B #1	28.67	7.48	21.19	1.99	0.251739	0.257956	0.034606
	28.41	7.45	20.96	1.76	0.295248		
	28.46	7.12	21.34	2.14	0.22688		
Ang II siGSK3B #2	28.57	7.63	20.94	1.74	0.29937	0.288767	0.014938
	28.75	7.67	21.08	1.88	0.271684		
	28.78	7.82	20.96	1.76	0.295248		

2. ET1

sample ID	ct target	ct 18s	dct	ddct	ratio	average	SD
SCR	24.48	7.38	17.1	1.57	0.336808	0.294669	0.03669
	24.51	7.09	17.42	1.89	0.269807		
	24.59	7.21	17.38	1.85	0.277392		
Ang II SCR	22.67	7.14	15.53	0	1	1.020736	0.080494
	22.95	7.35	15.6	0.07	0.952638		
	22.75	7.37	15.38	-0.15	1.109569		
Ang II siGSK3B #1	24.04	7.48	16.56	1.03	0.48971	0.485955	0.051911
	23.88	7.45	16.43	0.9	0.535887		
	23.86	7.12	16.74	1.21	0.432269		

Ang II siGSK3B #2	24.25	7.63	16.62	1.09	0.469761	0.489952	0.01881
	24.18	7.67	16.51	0.98	0.50698		
	24.37	7.82	16.55	1.02	0.493116		

Aug 23 2015

Sample ID	Absorbance	concentration (pg/ml)	average	SD
SCR	0.594	0.60	0.70	0.09614
	0.622	0.71		
	0.643	0.79		
Ang II SCR	1.338	3.51	3.65	0.54478
	1.472	4.03		
	1.275	3.26		
Ang II siGSK3B #1	0.884	1.74	1.48	0.22515
	0.802	1.41		
	0.773	1.30		
Ang II siGSK3B #2	0.726	1.12	1.32	0.20156
	0.781	1.33		
	0.829	1.52		

Real-time PCR performed Oct 12 2015
Samples prepared Oct 12 2015
EAhy926

1. ET1

sample ID	ct target	ct 18s	dct	ddct	ratio	average	SD
Vehicle	23.85	6.71	17.14	1.43	0.371131	0.341512	0.028512
	24.22	6.95	17.27	1.56	0.339151		
	24.03	6.65	17.38	1.67	0.314253		
Ang II	22.15	6.44	15.71	0	1	1.035987	0.099713
	22.19	6.68	15.51	-0.2	1.148698		
	22.34	6.57	15.77	0.06	0.959264		
Ang II LY2090314 5μM	23.17	6.88	16.29	0.58	0.668964	0.703281	0.047785
	23.18	6.92	16.26	0.55	0.68302		
	23.25	7.14	16.11	0.4	0.757858		
Ang II LY2090314 10μM	24.05	7.33	16.72	1.01	0.496546	0.492271	0.020706
	23.82	7.02	16.8	1.09	0.469761		
	23.84	7.16	16.68	0.97	0.510506		

Oct 15 2015

Sample ID	Absorbance	concentration (pg/ml)	average	SD
Vehicle	0.611	0.67	0.77	0.12908
	0.629	0.74		
	0.675	0.92		
Ang II	1.446	3.93	3.17	0.18251
	1.219	3.05		
	1.285	3.30		
Ang II LY2090314 5μM	1.029	2.30	2.11	0.19789
	0.928	1.91		
	0.984	2.13		
Ang II LY2090314 10μM	0.835	1.54	1.39	0.18882
	0.811	1.45		
	0.742	1.18		

Oct 7 2015
Eahy.926
ET1 promoter-LUC

Sample ID	raw luc activity	protein in μg per 20μl	GFP in ng per 20μl	luc activity per μg protein per ng GFP	ratio	average	S.D
control	318	23.29702	1830.46	0.007457041	1.196407	1.042871	0.137218
	276	25.75456	1844.4	0.005810317	0.932207		
	329	27.11986	1946.35	0.006232862	1		
Ang II	726	26.02762	2038.62	0.013682513	2.195222	2.289637	0.169289
	770	26.02762	1909.98	0.015489145	2.485077		
	731	26.30434	2037.2	0.013641317	2.188612		
Ang II GSK3 CA L	1053	28.84924	1904.29	0.019167299	3.0752	3.010608	0.100435
	868	24.75334	1837.52	0.019083316	3.061726		
	975	28.94026	1867.16	0.018043495	2.894897		
Ang II GSK3 CA M	1275	29.03128	1936.37	0.022680658	3.638883	3.540805	0.084983
	1179	28.12108	1924.89	0.021780904	3.494527		
	1240	28.94026	1970.29	0.021746487	3.489005		
Ang II GSK3 CA H	1683	27.48394	1976.04	0.030989131	4.971894	4.997484	0.26323
	1439	25.29946	1730.77	0.032863225	5.272574		
	1395	24.84436	1897.36	0.029593522	4.747983		
Ang II GSK3 DN L	624	26.75578	1846.45	0.012630758	2.026478	2.072014	0.219117
	649	24.93538	1807.46	0.014399918	2.310322		
	662	30.76066	1837.35	0.011713062	1.879243		
Ang II GSK3 DN M	514	29.03128	1842.69	0.009608258	1.541548	1.531483	0.043023
	506	28.12108	1840.45	0.00977675	1.568581		
	525	29.39536	1930.48	0.009251565	1.484321		
Ang II GSK3 DN H	472	31.30678	1852.09	0.008140321	1.306033	1.272002	0.074995
	468	30.39658	1865.79	0.008251984	1.323948		
	432	30.85168	1894.19	0.007392331	1.186025		

Oct 14 2015
Eahy.926
ET1 promoter-LUC WT and SRF MT

Sample ID	raw luc activity	protein in μg per 20μl	GFP in ng per 20μl	luc activity per μg protein per ng GFP	ratio	average	S.D
WT	462	22.8310796	2342.88	0.008637051	1	0.9939	0.051291
	539	25.2394688	2630.83	0.008117378	0.939832		
	551	24.5774628	2491.36	0.008998665	1.041868		
WT Ang II	1096	23.5070676	2496.33	0.018677128	2.162443	2.107077	0.138202
	1157	24.5070676	2474.45	0.019079339	2.209011		
	1132	25.7782532	2607.61	0.016840319	1.949776		
WT Ang II GSK3 CA	2078	26.2722552	2383.28	0.033187392	3.842445	3.676471	0.168714
	1964	26.2582732	2352.05	0.031800121	3.681826		
	1835	25.3614548	2389.96	0.030274102	3.505143		
WT Ang II GSK3 DN	778	24.4506544	2478.58	0.012837669	1.486349	1.404442	0.073191
	725	27.5586584	2263.85	0.0116207	1.345448		
	703	23.3614548	2521.91	0.011932347	1.38153		
MT	508	23.9342612	2259.35	0.009394208	1.087664	0.970319	0.121052
	547	25.7934708	2512.06	0.008442042	0.977422		
	432	24.3474728	2428.62	0.007305842	0.845872		
MT Ang II	538	26.2206644	2363.65	0.008680713	1.005055	1.040574	0.083495
	559	24.4366724	2331.54	0.009811307	1.135956		
	512	24.1454468	2503.39	0.008470445	0.98071		
MT Ang II GSK3 CA	661	28.4506544	2358.64	0.009850257	1.140465	1.066561	0.123548
	584	27.5586584	2655.52	0.007980042	0.923931		
	698	28.8074528	2471.04	0.009805524	1.135286		
MT Ang II GSK3 DN	545	26.6806444	2370.25	0.008617991	0.997793	0.988922	0.066818
	559	25.7886484	2388.21	0.009076339	1.050861		
	546	26.2346464	2624.56	0.007929776	0.918112		

Dec 7 2015
Eahy.926
ET1 promoter-LUC

Sample ID	raw luc activity	protein in μg per 20μl	GFP in ng per 20μl	luc activity per μg protein per ng GFP	ratio	average	S.D
control	475	20.7343478	2288.07	0.010012302	1.148174	1.040246	0.09447
	449	20.9215584	2530.51	0.008480945	0.972563		
	406	19.1366754	2432.95	0.008720198	1		
Ang II	952	21.1645818	2248.26	0.020006942	2.294322	2.592625	0.266647
	1179	21.1645818	2387.46	0.023332862	2.675726		
	1283	24.4108626	2146.58	0.024484794	2.807825		
Ang II SRF S224D	1869	23.6758236	2380.32	0.033164149	3.803142	3.867187	0.168937
	1791	22.0304726	2296.94	0.035393387	4.058782		
	1965	24.7568314	2433.95	0.032610379	3.739637		
Ang II SRF S224A	671	23.8378392	2420.42	0.011629603	1.33364	1.451046	0.117745
	868	24.0277612	2640.11	0.013683097	1.569127		
	740	23.7568314	2462.85	0.012647516	1.45037		

Real-time PCR performed Mar 29 2016
Samples prepared Mar 29 2016
EAhy926

1. ET1

sample ID	ct target	ct 18s	dct	ddct	ratio	average	SD
SCR	25.64	8.78	16.86	1.53	0.346277	0.337517	0.026462
	25.65	8.62	17.03	1.7	0.307786		
	25.76	8.95	16.81	1.48	0.358489		
SCR Ang II	23.94	8.42	15.52	0.19	0.876606	0.96824	0.080593
	23.96	8.67	15.29	-0.04	1.028114		
	24.02	8.69	15.33	0	1		
siSRF Ang II	25.46	8.82	16.64	1.31	0.403321	0.457539	0.046984
	25.28	8.91	16.37	1.04	0.486327		
	25.32	8.94	16.38	1.05	0.482968		
siSRF Ang II SRF S224D	23.45	8.34	15.11	-0.22	1.164734	1.262596	0.100769
	23.44	8.56	14.88	-0.45	1.36604		
	23.62	8.62	15	-0.33	1.257013		
siSRF Ang II SRF S224A	25.26	8.91	16.35	1.02	0.493116	0.456069	0.041139
	25.31	8.87	16.44	1.11	0.463294		
	25.34	8.73	16.61	1.28	0.411796		

Mar 25 2016

Sample ID	Absorbance	concentration (pg/ml)	average	SD
SCR	0.617	0.69	0.79	0.28468
	0.724	1.11		
	0.585	0.57		
Ang II SCR	1.409	3.79	3.27	0.53887
	1.134	2.71		
	1.287	3.31		
Ang II siSRF	0.863	1.65	1.53	0.11013
	0.808	1.44		
	0.825	1.50		
Ang II siSRF SRF S224D	1.572	4.43	4.61	0.27776
	1.585	4.48		
	1.701	4.93		
Ang II siSRF SRF S224A	0.882	1.73	1.62	0.09274
	0.849	1.60		
	0.836	1.55		

Real-time PCR performed April 5 2016
Samples prepared April 5 2016
EAhy926

1. ET1

sample ID	ct target	ct 18s	dct	ddct	ratio	average	SD
siSRF Ang II	26.22	9.83	16.39	1.47	0.360982	0.352717	0.028637
	26.19	9.86	16.33	1.41	0.376312		
	26.08	9.52	16.56	1.64	0.320856		
siSRF Ang II SRF	24.85	10.04	14.81	-0.11	1.079228	1.001939	0.076339
	24.66	9.74	14.92	0	1		
	24.76	9.73	15.03	0.11	0.926588		
siSRF Ang II SRF siGSK3b	26.07	9.88	16.19	1.27	0.41466	0.44914	0.047622
	25.83	9.92	15.91	0.99	0.503478		
	25.79	9.65	16.14	1.22	0.429283		
siSRF Ang II SRF S224D	24.51	10.08	14.43	-0.49	1.404445	1.53832	0.153115
	24.42	10.27	14.15	-0.77	1.70527		
	24.68	10.35	14.33	-0.59	1.505247		
siSRF Ang II SRF S224D siGSK3b	24.27	9.92	14.35	-0.57	1.484524	1.493118	0.231778
	24.06	9.93	14.13	-0.79	1.729074		
	24.36	9.78	14.58	-0.34	1.265757		

April 6 2016

Sample ID	Absorbance	concentration (pg/ml)	average	SD
siSRF Ang II	0.589	0.58	0.76	0.16612
	0.642	0.79		
	0.673	0.91		
siSRF Ang II SRF	1.323	3.45	2.98	0.47129
	1.205	2.99		
	1.082	2.51		
siSRF Ang II SRF siGSK3b	0.771	1.29	1.45	0.17327
	0.859	1.64		
	0.806	1.43		
siSRF Ang II SRF S224D	1.664	4.79	4.47	0.33556
	1.591	4.50		
	1.493	4.12		
siSRF Ang II SRF S224D siGSK3	1.564	4.39	4.31	0.37635
	1.438	3.90		
	1.627	4.64		

Real-time PCR performed April 5 2016
Samples prepared April 5 2016
EAhy926

1. ET1

sample ID	ct target	ct 18s	dct	ddct	ratio	average	SD
siSRF Ang II	24.59	8.59	16	1.43	0.371131	0.362507	0.027074
	24.88	8.72	16.16	1.59	0.332171		
	24.61	8.66	15.95	1.38	0.384219		
siSRF Ang II SRF	24.07	9.49	14.58	0.01	0.993092	1.016704	0.035084
	24.24	9.75	14.49	-0.08	1.057018		
	24.15	9.58	14.57	0	1		
siSRF Ang II SRF LY2090314	25.33	9.98	15.35	0.78	0.582367	0.518722	0.05996
	25.26	9.72	15.54	0.97	0.510506		
	25.49	9.81	15.68	1.11	0.463294		
siSRF Ang II SRF S224D	23.89	9.89	14	-0.57	1.484524	1.454695	0.131941
	24.19	10.01	14.18	-0.39	1.310393		
	24.06	10.14	13.92	-0.65	1.569168		
siSRF Ang II SRF S224D LY2090314	24.27	10.27	14	-0.57	1.484524	1.394378	0.108081
	24.58	10.36	14.22	-0.35	1.274561		
	24.55	10.49	14.06	-0.51	1.42405		

April 11 2016

Sample ID	Absorbance	concentration (pg/ml)	average	SD
siSRF Ang II	0.677	0.93	0.86	0.16486
	0.691	0.98		
	0.612	0.67		
siSRF Ang II SRF	1.245	3.15	3.13	0.23144
	1.296	3.35		
	1.178	2.88		
siSRF Ang II SRF LY2090314	0.891	1.76	1.71	0.13829
	0.902	1.81		
	0.836	1.55		
siSRF Ang II SRF S224D	1.572	4.43	4.27	0.14043
	1.504	4.16		
	1.518	4.21		
siSRF Ang II SRF S224D LY2090314	1.527	4.25	4.19	0.22457
	1.561	4.38		
	1.449	3.94		

Real-time PCR performed May 1 2016
Samples prepared May 1 2016

1. ET1 promoter

sample ID	ct target	dct	ratio	average	SD
SCR input	23.19	23.135			
	23.08				
SCR SRF	28.77	5.635	0.201232	0.181264	0.020016
	29.09	5.955	0.1612		
	28.92	5.785	0.18136		
SCR MRTF-A	29.61	6.475	0.112417	0.101598	0.009423
	29.82	6.685	0.097188		
	29.85	6.715	0.095188		
SCR H3K4M3	29.31	6.175	0.138401	0.166941	0.028004
	29.03	5.895	0.168046		
	28.82	5.685	0.194377		
SCR H3K9M2	28.12	4.985	0.315766	0.323858	0.037991
	28.24	5.105	0.290564		
	27.91	4.775	0.365243		
SCR BRG1	30.37	7.235	0.066382	0.075331	0.00814
	30.15	7.015	0.077317		
	30.06	6.925	0.082294		
SCR SET1	30.32	7.185	0.068723	0.062192	0.009729
	30.36	7.225	0.066843		
	30.75	7.615	0.05101		
SCR JMJD1A	30.43	7.295	0.063678	0.052846	0.009382
	30.86	7.725	0.047265		
	30.85	7.715	0.047594		
SCR Ang II input	22.74	22.63			
	22.52				
SCR Ang II SRF	26.98	4.35	0.490365	0.435096	0.05418
	27.16	4.53	0.432847		
	27.34	4.71	0.382075		
SCR Ang II MRTF-A	27.56	4.93	0.328036	0.392954	0.05624
	27.19	4.56	0.423939		
	27.18	4.55	0.426888		
SCR Ang II H3K4M3	26.31	3.68	0.780207	0.963456	0.160748
	25.84	3.21	1.080672		
	25.91	3.28	1.029489		
SCR Ang II H3K9M2	28.89	6.26	0.130482	0.117791	0.020447
	28.91	6.28	0.128686		
	29.36	6.73	0.094204		
SCR Ang II BRG1	27.85	5.22	0.268302	0.302205	0.040286
	27.73	5.1	0.291573		
	27.48	4.85	0.34674		
SCR Ang II SET1	27.78	5.15	0.281641	0.279039	0.045837
	28.06	5.43	0.231957		

	27.58	4.95	0.32352		
SCR Ang II JMJD1A	28.25	5.62	0.203335	0.24916	0.039865
	27.81	5.18	0.275845		
	27.85	5.22	0.268302		
siGSK3b Ang II input	22.65	22.82			
	22.99				
siGSK3b Ang II SRF	27.66	4.84	0.349152	0.420348	0.061678
	27.28	4.46	0.454366		
	27.27	4.45	0.457527		
siGSK3b Ang II MRTF-A	28.71	5.89	0.168629	0.161993	0.010502
	28.72	5.9	0.167465		
	28.88	6.06	0.149885		
siGSK3b Ang II H3K4M3	28.16	5.34	0.246888	0.279396	0.028227
	27.91	5.09	0.293601		
	27.89	5.07	0.297699		
siGSK3b Ang II H3K9M2	28.17	5.35	0.245183	0.285778	0.035221
	27.86	5.04	0.303955		
	27.84	5.02	0.308198		
siGSK3b Ang II BRG1	29.12	6.3	0.126914	0.131258	0.008293
	29.13	6.31	0.126038		
	28.97	6.15	0.14082		
siGSK3b Ang II SET1	29.44	6.62	0.101667	0.11285	0.009992
	29.19	6.37	0.120904		
	29.25	6.43	0.115978		
siGSK3b Ang II JMJD1A	29.55	6.73	0.094204	0.094834	0.010849
	29.71	6.89	0.084315		
	29.38	6.56	0.105985		

Real-time PCR performed May 20 2016
Samples prepared May 20 2016

1. ET1 promoter

sample ID	ct target	dct	ratio	average	SD
input	22.74	22.565			
	22.39				
SRF	27.84	5.275	0.258266	0.247292	0.022203
	27.82	5.255	0.261871		
	28.06	5.495	0.221738		
MRTF-A	28.55	5.985	0.157883	0.163208	0.021098
	28.31	5.745	0.186459		
	28.67	6.105	0.145282		
H3K4M3	27.66	5.095	0.292585	0.255424	0.032276
	27.95	5.385	0.239306		
	27.98	5.415	0.234381		
H3K9M2	27.51	4.945	0.324643	0.371915	0.040962
	27.22	4.655	0.396922		
	27.23	4.665	0.39418		
BRG1	29.77	7.205	0.067776	0.073823	0.010881
	29.78	7.215	0.067308		
	29.42	6.855	0.086385		
SET1	29.41	6.845	0.086986	0.081169	0.006826
	29.48	6.915	0.082866		
	29.65	7.085	0.073655		
JMJD1A	29.88	7.315	0.062801	0.060117	0.007085
	29.82	7.255	0.065468		
	30.15	7.585	0.052082		
Ang II input	22.52	22.44			
	22.36				
Ang II SRF	26.31	3.87	0.683934	0.622845	0.093882
	26.34	3.9	0.669858		
	26.72	4.28	0.514744		
Ang II MRTF-A	26.51	4.07	0.595399	0.509355	0.079409
	26.78	4.34	0.493776		
	26.95	4.51	0.438889		
Ang II H3K4M3	25.84	3.4	0.947323	1.021392	0.139738
	25.86	3.42	0.934281		
	25.52	3.08	1.182572		
Ang II H3K9M2	29.06	6.62	0.101667	0.112355	0.010469
	28.91	6.47	0.112807		
	28.79	6.35	0.122591		
Ang II BRG1	27.34	4.9	0.334929	0.375562	0.060438
	27.29	4.85	0.34674		
	26.93	4.49	0.445016		
Ang II SET1	27.35	4.91	0.332616	0.342971	0.032547
	27.42	4.98	0.316862		

	27.16	4.72	0.379436		
Ang II JMJD1A	27.44	5	0.3125	0.373433	0.053065
	27.09	4.65	0.3983		
	27.05	4.61	0.409498		
LY209 Ang II input	22.21	22.335			
	22.46				
LY209 Ang II SRF	26.49	4.155	0.561333	0.616021	0.078209
	26.44	4.105	0.581128		
	26.16	3.825	0.705603		
LY209 Ang II MRTF-A	27.75	5.415	0.234381	0.214157	0.017571
	27.96	5.625	0.202631		
	27.94	5.605	0.20546		
LY209 Ang II H3K4M3	27.18	4.845	0.347944	0.382443	0.030005
	26.99	4.655	0.396922		
	26.97	4.635	0.402463		
LY209 Ang II H3K9M2	27.49	5.155	0.280666	0.329574	0.042373
	27.16	4.825	0.352801		
	27.15	4.815	0.355255		
LY209 Ang II BRG1	28.81	6.475	0.112417	0.123379	0.011616
	28.69	6.355	0.122167		
	28.54	6.205	0.135553		
LY209 Ang II SET1	28.22	5.885	0.169215	0.172653	0.018898
	28.34	6.005	0.155709		
	28.03	5.695	0.193034		
LY209 Ang II JMJD1A	28.29	5.955	0.1612	0.159416	0.01433
	28.45	6.115	0.144278		
	28.19	5.855	0.17277		

Real-time PCR performed June 24 2016
Samples prepared June 24 2016

1. ET1 promoter

sample ID	ct target	dct	ratio	average	SD
SRF WT input	22.75	22.595			
	22.44				
SRF WT FLAG MRTF-A	32.17	9.575	0.013111	0.014017	0.001648
	31.89	9.295	0.015919		
	32.18	9.585	0.01302		
SRF WT FLAG BRG1	33.64	11.045	0.004733	0.005123	0.00079
	33.68	11.085	0.004603		
	33.29	10.695	0.006032		
SRF WT FLAG SET1	34.07	11.475	0.003513	0.004057	0.000504
	33.71	11.115	0.004509		
	33.83	11.235	0.004149		
SRF WT FLAG JMJD1A	33.56	10.965	0.005003	0.004239	0.000662
	33.92	11.325	0.003898		
	33.95	11.355	0.003818		
SRF WT FLAG IgG	34.08	11.485	0.003489	0.004126	0.000656
	33.62	11.025	0.004799		
	33.85	11.255	0.004092		
SRF WT Ang II input	22.38	22.445			
	22.51				
SRF WT Ang II FLAG MRTF-A	29.93	7.485	0.05582	0.060131	0.009163
	29.98	7.535	0.053919		
	29.59	7.145	0.070655		
SRF WT Ang II FLAG BRG1	30.71	8.265	0.032508	0.026979	0.004795
	31.12	8.675	0.024466		
	31.15	8.705	0.023963		

SRF WT Ang II FLAG SET1	31.24	8.795	0.022513	0.020566	0.003239
	31.25	8.805	0.022358		
	31.66	9.215	0.016827		
SRF WT Ang II FLAG JMJD1A	31.19	8.745	0.023307	0.026405	0.003878
	31.08	8.635	0.025154		
	30.79	8.345	0.030754		
SRF WT Ang II FLAG IgG	33.85	11.405	0.003688	0.004206	0.000602
	33.45	11.005	0.004866		
	33.71	11.265	0.004063		
SRF S234A input	22.59	22.655			
	22.72				
SRF S234A FLAG MRTF-A	33.13	10.475	0.007026	0.008011	0.000865
	32.83	10.175	0.00865		
	32.88	10.225	0.008355		
SRF S234A FLAG BRG1	34.05	11.395	0.003713	0.004104	0.000632
	34.03	11.375	0.003765		
	33.67	11.015	0.004832		
SRF S234A FLAG SET1	33.82	11.165	0.004355	0.004445	0.000623
	33.59	10.935	0.005108		
	33.99	11.335	0.003871		
SRF S234A FLAG JMJD1A	33.92	11.265	0.004063	0.003921	0.000405
	34.15	11.495	0.003465		
	33.86	11.205	0.004236		
SRF S234A FLAG IgG	33.71	11.055	0.0047	0.003869	0.000725
	34.12	11.465	0.003537		
	34.19	11.535	0.00337		
SRF S234A Ang II input	22.41	22.585			
	22.76				
SRF S234A Ang II FLAG MRTF-A	32.24	9.655	0.012404	0.014822	0.002106
	31.85	9.265	0.016254		

	31.89	9.305	0.015809		
SRF S234A Ang II FLAG BRG1	32.71	10.125	0.008955	0.007546	0.001264
	33.03	10.445	0.007174		
	33.17	10.585	0.00651		
SRF S234A Ang II FLAG SET1	33.25	10.665	0.006159	0.007179	0.000885
	32.94	10.355	0.007635		
	32.92	10.335	0.007742		
SRF S234A Ang II FLAG JMJD1A	32.66	10.075	0.009271	0.007758	0.001312
	33.05	10.465	0.007075		
	33.08	10.495	0.006929		
SRF S234A Ang II FLAG IgG	34.09	11.505	0.003441	0.003829	0.000359
	33.91	11.325	0.003898		
	33.82	11.235	0.004149		

Figure 1C

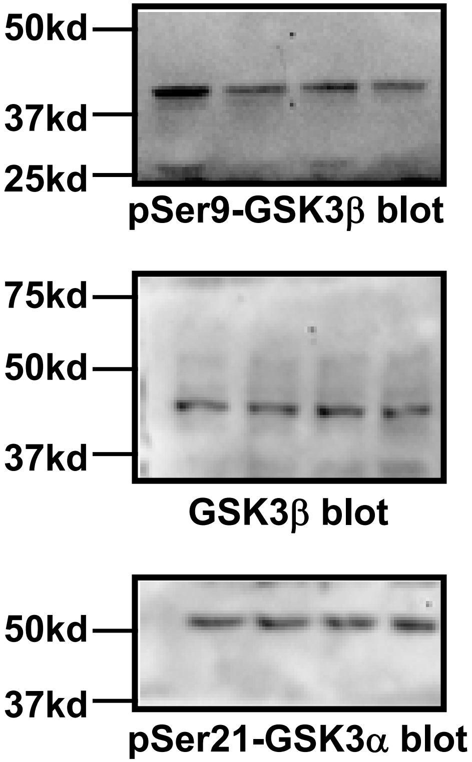

Figure 3A

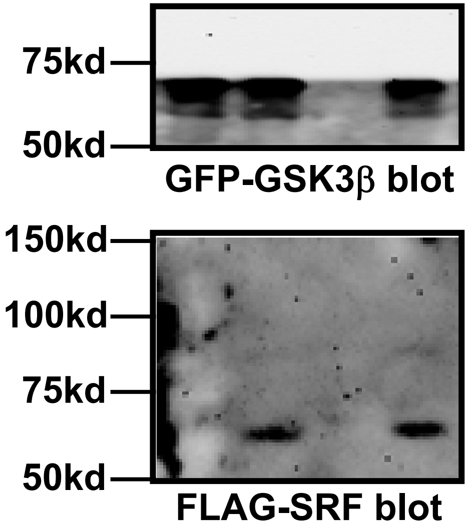

Figure 3B

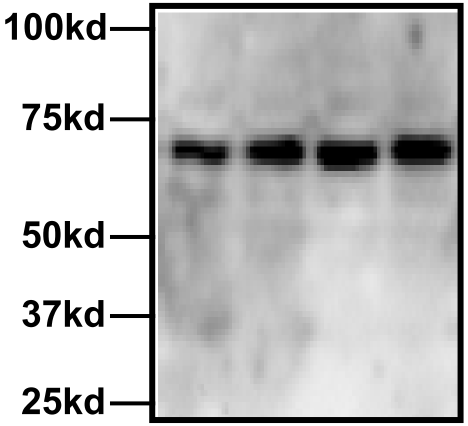

phospho-SRF blot

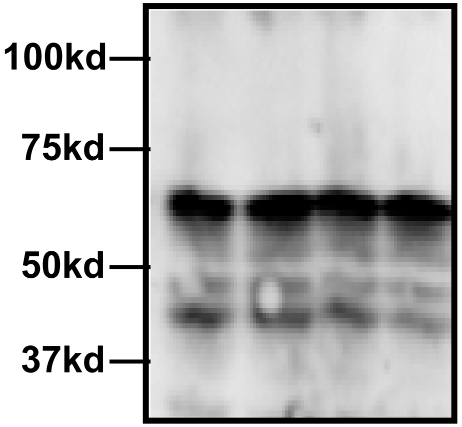

SRF blot

Figure 3C

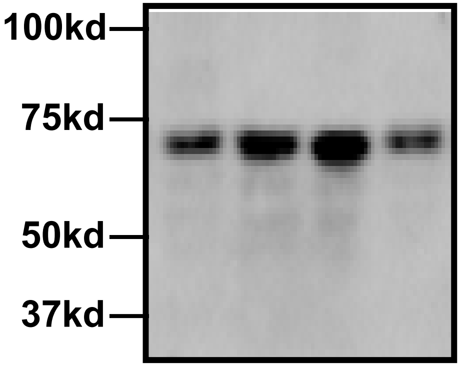

phospho-SRF blot

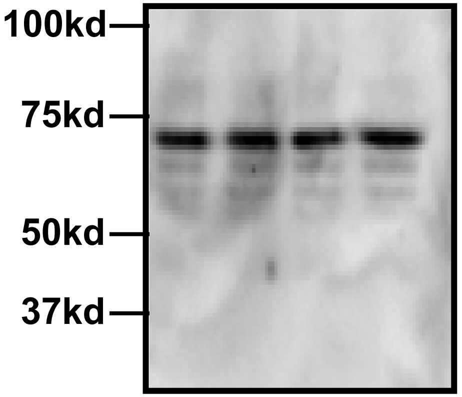

SRF blot

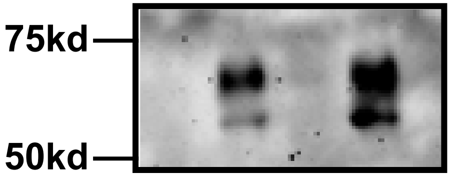

GFP-GSK3β blot

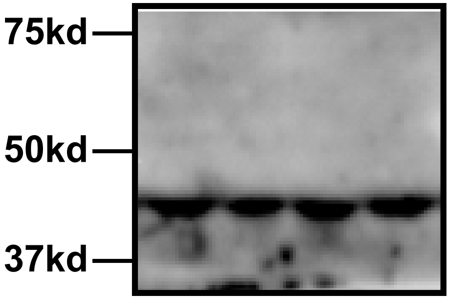

β-actin blot

Figure 3D

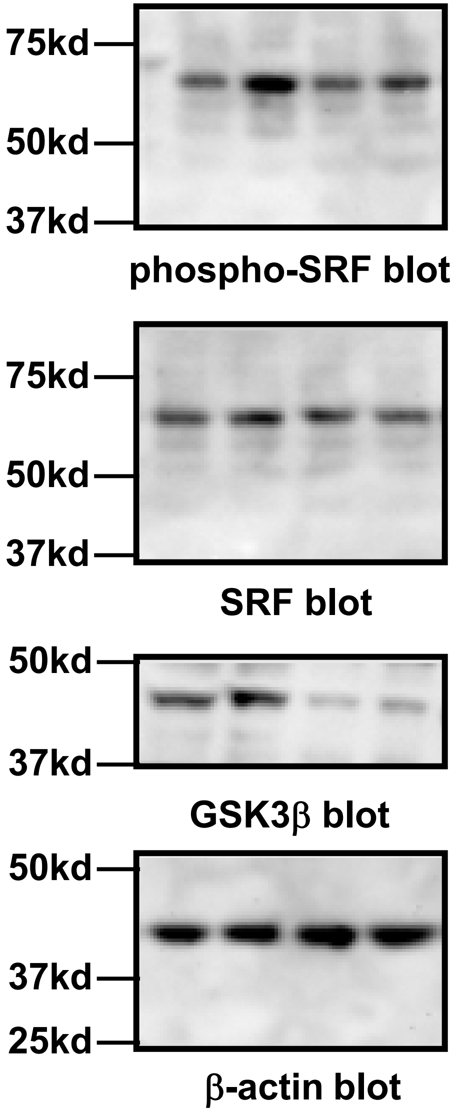

Figure 3E

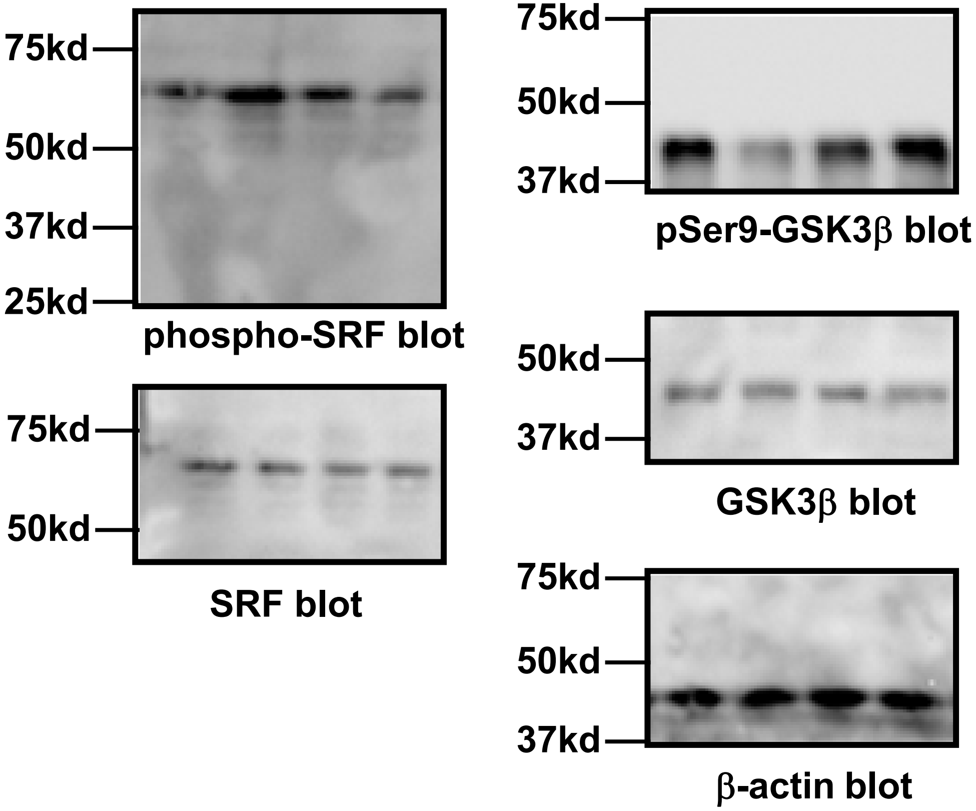

Figure 4B

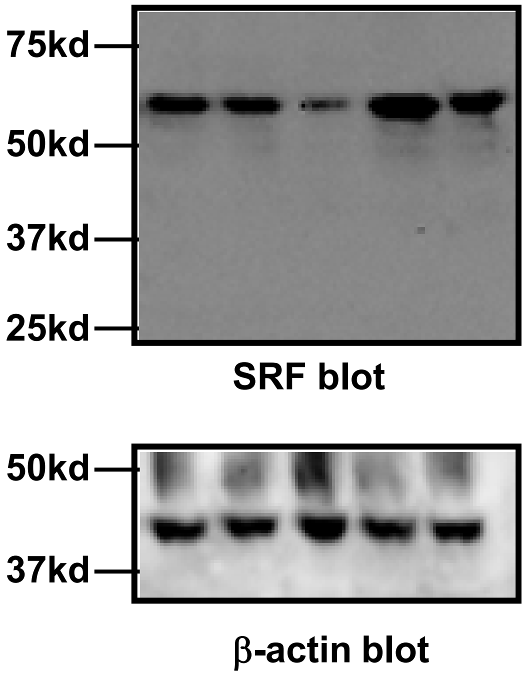

Supplement: Supplementary file 1 [file Data_Sheet_1.PDF]
